# Pharmacological treatment of cancer pain and opioid induced nausea and vomiting: online survey and comparison with current guidelines

**DOI:** 10.1007/s00520-024-08628-7

**Published:** 2024-06-15

**Authors:** Jan Gaertner, Christopher Boehlke, Gudrun Kreye, Tanja Fusi-Schmidhauser, Evelyn Mueller, Carmen Roch

**Affiliations:** 1 Palliative Care Center Basel, Basel, Switzerland; 2 Department of Clinical Research, University Hospital Basel and University of Basel, Basel, Switzerland; 3 Division of Palliative Care, Department of Internal Medicine, Karl Landsteiner University of Health Sciences, Krems, Austria; 4 Karl Landsteiner University of Health Sciences, University Hospital, Krems, Austria; 5 Palliative and Supportive Care Clinic and Department of Internal Medicine, Ente Ospedaliero Cantonale, Lugano, Switzerland; 6 Department of Rehabilitation and Geriatrics, University of Geneva, Geneva, Switzerland; 7 Interdisciplinary Center for Palliative Medicine, University Hospital Wuerzburg, Würzburg, Germany

**Keywords:** Pharmacological symptom control, Palliative care, Pain management, Opioid induced nausea and vomiting, Survey

## Abstract

**Purpose:**

We assumed that in Palliative Care, even in common clinical situations, the choice of drugs differs substantially between physicians. Therefore, we assessed the practice of pharmaceutical treatment choices of physicians for cancer pain and opioid-induced nausea and vomiting (OINV) and the rationale for their choices.

**Methods:**

An online survey was conducted with physicians covering the following domains: i) Cancer pain therapy: non-opioids in addition to opioids: choice of drug ii) prevention of OINV: choice of drug and mode of application. Current guidelines concerning cancer pain therapy and prevention of OINV were compared.

**Results:**

Two-hundred-forty European physicians responded to our survey. i) Use of non-opioids in addition to opioids for the treatment of cancer pain: Only 1.3% (n = 3) of respondents never used an additional non-opioid. Others mostly used: dipyrone/metamizole (49.2%, n = 118), paracetamol/acetaminophen (34.2%, n = 82), ibuprofen / other NSAIDs (11.3%, n = 27), specific Cox2-inhibitors (2.1%, n = 5), Aspirin (0.4%, n = 1), no answer (2.9%, n = 7). ii) Antiemetics to prevent OINV: The drugs of choice were metoclopramide (58.3%, n = 140), haloperidol (26.3%, n = 63), 5-HT3 antagonists (9.6%, n = 23), antihistamines (1.3%, n = 3) and other (2.9%, n = 7); no answer (1.7%, n = 4). Most respondents prescribed the substances on-demand (59.6%, n = 143) while others (36.3%, n = 87) provided them as around the clock medication. Over both domains, most physicians answered that their choices were not based on solid evidence from randomized controlled trials (RCTs). Guidelines were inconsistent regarding if and what non-opioid to use for cancer pain and recommend anti-dopaminergic drugs for prevention or treatment of OINV.

**Conclusions:**

Physician’s practice in palliative care for the treatment of cancer pain and OINV differed substantially. Respondents expressed the lack of high-quality evidence- based information from RCTs. We call for evidence from methodologically high-quality RCTs to be available to inform physicians about the benefits and harms of pharmacological treatments for common symptoms in palliative care.

**Supplementary Information:**

The online version contains supplementary material available at 10.1007/s00520-024-08628-7.

## Purpose

The WHO demands impeccable assessment and treatment of pain and other problems for Palliative Care (PC) [1]. Many different strategies for treating pain are available: not only pharmacological approaches, but also complementary or even interventional measures [2–4]. Within these options, drug-based pain therapy is the quickest, easiest and most common therapy to implement [5]. “Impeccability” in the context of pharmacological treatment for symptom control implies to recommend and utilize only effective and safe medication. Otherwise, there is large potential for harm in a vulnerable patient population.

Pain is one of the most common symptoms in cancer patients, and the more advanced the disease, the more frequently adequate pain treatment is required [6, 7]. Nearly half of patients diagnosed with a solid tumor will suffer from moderate or severe cancer pain. However, due to several barriers, current pain management may be ineffective. For example, there is an insufficient evidence base for many of the pharmacological treatment options for cancer pain, leading to more than 40% of the patients being treated insufficiently [8]. According to the WHO analgesic ladder, the combination of opioids with non-opioids is recommended for moderate to severe pain in the context of cancer (WHO guidelines on cancer pain) [9, 10]. However, recommendations on which non-opioid adjacent to opioids to use are lacking, possibly leading to a variety of pain management regimes with unknown benefits and risks.

Other common problems in PC symptom control include the use of an antiemetic prophylaxis of opioid-induced nausea and vomiting (OINV) [11]. On one hand the patients’ benefit is unclear, but on the other hand a variety of side effects (extrapyramidal, tiredness, constipation, dry mouth, QT-prolongation) are well-known [12].

We hypothesize that “impeccability” may rely largely on personal judgment and vague or inhouse recommendation. We assume that as a result of the above, in PC, even in common clinical situations, the choice of drugs differs substantially between different physicians.

## Methods

We conducted an online survey of professionals in general and specialist palliative care and related medical fields (survey questions in Supplement) and compared recommendations of major palliative care guidelines.

### Survey development

Survey items for the main research questions were developed based on literature and guidelines. The newly developed items were pre-tested regarding face validity, suitability of multiple-choice questions, and comprehensibility through three cognitive interviews with physicians, and modified by a team of two experts in the field (palliative care and pharmacology). The survey comprised: (a) sociodemographic and occupational characteristics of participants; (b) the specially developed items on pharmacological choices and reasons for choices. The items employed multiple-choice as answer options and participants could include comments and suggestions in an open-ended question. Covered domains: i) cancer pain therapy: non-opioids in addition to opioids ii) prevention of OINV: choice of drug and mode of application.

### Sample and recruitment

We invited participants of the EAPC (European Association for Palliative Care) Congress 2023 to complete the survey by distributing flyers. Furthermore, all participants were invited to share the link of the survey in their professional network (i.e. Linkedin, Facebook and personal mailings). The survey was anonymous, no personal data were collected. All participants were informed that by participating and answering the questions they agreed to a publication of aggregated results. The online questionnaire was programmed by Smart-Q, a software provider from Bochum, Germany. The online-survey was accessible for 4 weeks in June and July 2023. We followed the CROSS checklist on reporting survey-based studies [13].

### Data analysis

Statistical analysis was performed using Microsoft® Excel (Version 16.80) and IBM SPSS (Version 29.0). The data is presented descriptively. Chi square tests, and in cases of cell frequencies below 5 Fisher’s exact test, were used to examine differences in drug prescription between specialists of palliative care and non-specialists. Standardised residuals of z < -1.96 or z > 1.96 were employed to determine cells with significant deviations between expected and actual cell frequencies (post-hoc test). Due to the explorative approach of the analysis, alpha level was not adjusted and was 5% (two-tailed) for all tests. Missing data were not imputed.

### Statement of ethics

Given the anonymous nature of the survey and the absence of patient health data, ethical approval for this survey involving healthcare professionals was not deemed necessary by the local ethics committee, as per institutional guidelines and regulations. The survey aimed to investigate prescription habits of physicians. Participation in the survey was voluntary, and respondents were informed about the purpose and confidentiality of their responses. No personally identifiable information was collected, ensuring participant anonymity and data confidentiality.

### Comparison of guidelines

We compared the recommendations of the palliative care guidelines of the European Association for Palliative Care (EAPC), the European Society for Medical Oncology (ESMO), American Society of Clinical Oncology (ASCO) and the German S-3 Guideline for Palliative Care for patients with incurable cancer. For analgesics, we compared the recommendations for the treatment of moderate to severe pain; for anti-emetics, we compared the recommendations for the treatment of opioid-induced nausea and vomiting.

## Results

### Sample

Two-hundred-forty physicians completed the survey. Due to the social-media-approach, a response rate could not be calculated. Almost half of respondents were palliative care specialists, the other respondents were from different specialties: oncology, cardiology, internal medicine, general practitioner and other disciplines. More than half of the respondents had more than 10 years of experience in their field. The majority of respondents were from Europe (Table 1).

**Table 1:** Specialties, level of experience and location of physicians

N = 240	n	%
**Best described discipline**		
Specialist palliative care	112	46.7
Oncology	45	18.8
Geriatric oncology	3	1.3
Cardiology	2	0.8
Internal medicine	38	15.8
General practitioner	14	5.8
Other disciplines	26	10.8
**Experience in that discipline**		
< 5 years	46	19.2
5–10 years	55	22.9
> 10 years	137	57.1
No answer	2	0.8
**Origin**		
Europe	234	97.5
*-Scandinavia*	*3*	*1.3*
*-Central Europe*	*219*	*91.3*
*-Southern Europe*	*8*	*3.3*
*-No answer*	*4*	*1.7*
North America	4	1.7
Asia	2	0.8

### Cancer pain therapy: which non-opioids are used in addition to opioids?

Most physicians used non-opioids adjunct to opioids in cancer pain therapy (WHO step III) regularly (65.8%, n = 158) or sometimes (26.7%, n = 64).

**Fig. 1:**
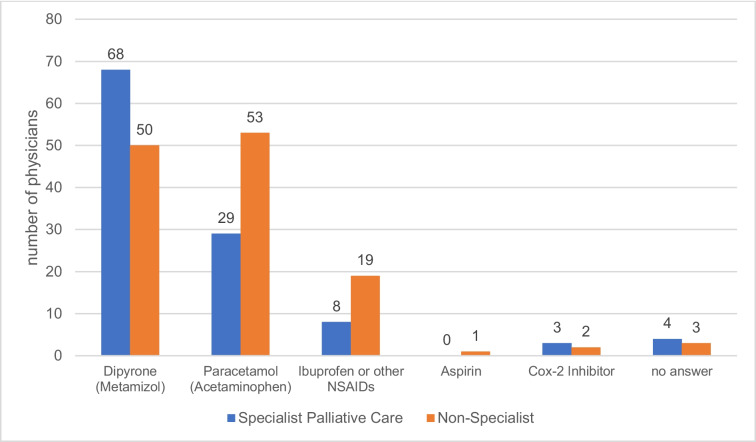
Non-opioids used adjunct to opioids in cancer pain therapy (n_s__pecialists_ = 112, n_non-specialists_ = 128)

The most frequently used non-opioids were dipyrone/metamizole (49.2%, n = 118), paracetamol/acetaminophen (34.2%, n = 82), ibuprofen / other NSAIDs (11.3%, n = 27), specific Cox2-inhibitors (2.1%, n = 5), and Aspirin (0.4%, n = 1). Only 1.3% (n = 3) of respondents never used an additional non-opioid; no answer (2.9%, n = 7) (Fig. 1).

A comparison of the group of specialist palliative care physicians with the group of other physicians showed significant differences in drug choice (p = 0.003, Cramér’s Phi V = 0.25). Specialists prescribe dipyrone/metamizole significantly more often (z = 3.5) than non-specialists and paracetamol less often (z = -2.5) (Fig. 1).

### What is the rationale for drug choice?

Forty-two percent of participants (n = 102) responded that their choices were based on national or international guidelines. A quarter of the physicians (25.4%, n = 61) reported that they decided based on experience or intuition. Twenty percent of the physicians (19.6%, n = 47) rely on inhouse standards, and 6% of physicians answered that they either rely on randomized controlled trials (RCTs; n = 14) or are unsure on what their decisions rely on (n = 13). One percent of physicians (1.3%, n = 3) did not provide any information. There were no significant group differences between specialist and non-specialist palliative care physicians.

Because most physicians stated they based their decision on national and international guidelines, we consulted the ESMO, ASCO, EAPC and German S3- guidelines concerning symptom control of moderate to severe cancer pain in palliative care to better understand the heterogeneity of prescription practice (Table 2).

**Table 2:** Comparison of major palliative care guidelines

**Treatment of moderate to severe cancer pain**				
	ESMO [14]	ASCO [15]	S3-guideline (Germany) [16]	EAPC [17]
Non-Opioids	Non-Opioids not mentioned	Non-Opioids not mentioned	Non-Opioids are recommended in conjunction with opioids: Dipyrone may be preferred to paracetamol and NSAIDs because of less adverse events	Non-Opioids "weak recommendation" in conjunction with opioids: Paracetamol should be preferred to NSAIDs because of a more favourable side-effect profile, but its efficacy is not well documented. Dipyrone not mentioned
Opioids	Strong opioids	Opioids (recommendation: strong, level of evidence: moderate)	Strong opioids (recommendation strong, level of evidence -1)	Strong opioids
**Symptom management of opioid-induced nausea and vomiting (OINV)**				
Drug	Metoclopramide, antidopaminergic drugs	Metoclopramide, prochlorperazine	Antidopaminergic drugs (e.g., haloperidol) and other drugs with antidopaminergic and additional modes of action (e.g., metoclopramide)	Antidopaminergic drugs (e.g., haloperidol) and other drugs with antidopaminergic and additional modes of action (e.g., metoclopramide)
Application mode	Not mentioned	Around the clock for the first few days of opioid therapy, with gradual weaning of the antiemetic; around the clock for patients reporting previous OINV	Not mentioned	Not mentioned

ESMO European Society for Medical Oncology, ASCO American Society for Clinical Oncology, EAPC European Association for Palliative Care

The major palliative care guidelines differ in their recommendations regarding symptom control of moderate to severe cancer pain. While the ESMO- and ASCO-guidelines do not at all mention the use of non-opioids adjunct to opioids for the treatment of moderate to severe cancer pain, the German S3-guideline and the EAPC-guideline recommend the use of non-opioids [14–16]. Although the S3-guideline is an adaptation of the EAPC-guideline for the pharmacological treatment of cancer pain for Germany and both guidelines offer evidence-based recommendations, the S3-guideline suggests considering dipyrone as non-opioid of choice, a substance which is not mentioned in the EAPC-guideline [16, 17]. In contrast, the EAPC-guideline recommends preferring paracetamol because of a more favourable side-effect profile (Table 2) [17].

### How is opioid-induced nausea and vomiting (OINV) prevented on initiation of opioid therapy in palliative care?

The drugs of choice were metoclopramide (58.3%, n = 140), haloperidol (26.3%, n = 63), 5-HT3 antagonists (9.6%, n = 23), antihistamines (1.3%, n = 3) and others (2.9%, n = 7); four physicians did not answer (1.7%, n = 4). Most respondents reported that they prescribe the substances on-demand (59.6%, n = 143) while other respondents provided them as around the clock medication (36.3%, n = 87). 8 (3.3%) stated simply other modes of application without specification. Two physicians (0.8%) provided no information.

**Fig. 2:**
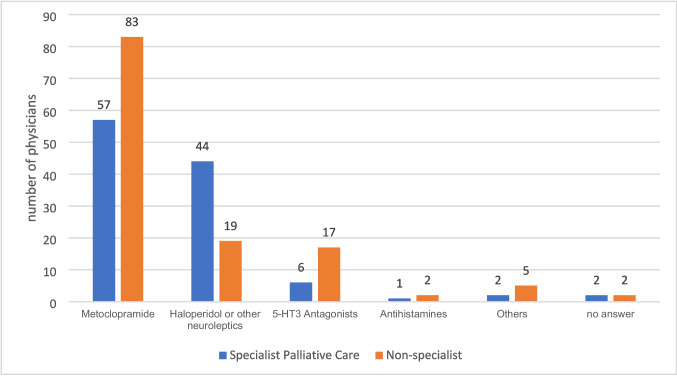
Choice of antiemetics (n_s__pecialists_ = 112, n_non-specialists_ = 128)

The choices of drugs (p < 0.001, Cramér’s Phi V = 0.31) and their provision “around the clock” or “on-demand” (p = 0.007, Cramér’s Phi V = 0.20) as well as the base of these decisions (i.e. national guidelines; p < 0.001, Cramér’s Phi V = 0.37) differed substantially between specialist palliative care physicians and other disciplines providing mainly general palliative care. Specialist palliative care physicians tended to prescribe haloperidol more often (z = 4.3) while they prescribed metoclopramide (z = -2.2) and 5-HT3 antagonists (z = -2.1) less frequently than non-specialist. At the same time, even among physicians from specialist palliative care the choice of drug was heterogenous (Fig. 2). Specialists prescribe antiemetics more frequently as taken “on-demand” than non-specialists (z = -3.1).

As a rationale for the decision on choice of antiemetics most physicians reported national or international guidelines (32.1%, n = 77), followed by intuition and experience (31.7%, n = 76), inhouse standards (27.9%, n = 67) or randomized controlled trials (2.5%, n = 6); 5.8% (n = 14) of participants reported to be ‘unsure’ or did not answer the question.

The recommendations of the guidelines are closely related and are essentially limited to antidopaminergic drugs. Only the ASCO guideline recommends on the mode of administration: “around the clock” medication for the first few days of opioid therapy and for patients reporting previous OINV; the other guidelines abstain from doing so (Table 2).

## Discussion

### *Cancer* pain therapy

This survey showed substantial heterogeneity in drug usage for common situations in PC: non-opioid use adjunct to opioids for cancer pain therapy and prophylaxis and treatment of OINV. We were able to show that most physicians prescribe non-opioid additives and reported to stick to guideline recommendations. There were significant differences not only between specialist palliative care physicians and other specialties, but also within the group of specialist palliative care physicians. We assume that the heterogeneity might stem from differences in the major guidelines on one hand, and on the other hand from different access to medication. While metamizole can easily be prescribed in Germany and some other European countries, it is not available in many other countries, including the USA and parts of Europe [18]. In addition, the use of dipyrone/metamizole is not possible in many countries due to regulatory restrictions and the debate about its use concerning its side effects is ongoing [19, 20].

Another apparent reason for the heterogeneity might be lacking or conflicting evidence concerning use of non-opioids for the treatment of cancer pain. According to the WHO analgesic ladder, moderate to severe cancer pain should be treated with opioids and possible addition of non-opioid analgesics [10]. However, a recently published RCT investigating the efficacy of acetaminophen (paracetamol) together with opioids in 112 randomized patients did not show a benefit of paracetamol when compared to placebo [21]. For other non-opioids like NSAIDs a Cochrane review concluded that there is no high‐quality evidence to prove or disprove that they are useful in treating people with cancer pain if used in addition to opioids [22] arguing that more high-quality studies were needed. Along these lines, a recently published survey concluded that a randomized controlled trial of NSAIDs as opioid adjuncts for cancer-related bone pain would be the most pragmatic design supported by palliative care clinicians to benefit clinical practice [23]. Accordingly, there is no robust evidence that investigates dipyrone/metamizole with opioids in cancer pain management [24]. In conclusion, the efficacy of non-opioid analgesics like dipyrone/metamizole and NSAIDS (ibuprofen) together with opioids in cancer pain management is unclear leading to a variety of potentially ineffective non-opioids used on the one hand and many patients not receiving non-opioid analgesics regularly on the other hand.

We were able to show that in the cohort of palliative care specialists dipyrone/metamizole was the predominantly used additive analgesic. This was also observed in the context of patient care in hospices in Germany [25]. As most participating physicians came from central Europe with a likely focus on Germany and Switzerland, this could support our thesis that either PC specialists often adhere to the guidelines in clinical practice (S3-guideline) or metamizole is easy to prescribe in Germany and Switzerland due to its availability. Beyond this, the German S3-guideline for PC is probably well known to palliative care specialists due to the focus of their work.

### OINV prevented on initiation of opioid therapy

As for cancer pain management the evidence-base for the prophylaxis or treatment of OINV is scarce, too, which may explain some the heterogeneity in drug applications. Cancer patients who started to receive oral oxycodone were randomly assigned to receive either prochlorperazine (dopamine receptor antagonist, not available in Germany) or placebo prophylactically [26]. There was no statistically significant benefit for patients treated with prochlorperazine. The authors propose that further research is needed to evaluate whether other antiemetics would be effective in preventing OINV in specific patient populations [26]. Furthermore, one trial investigated the efficiency of ondansetron and metoclopramide comparing it with placebo after onset (not prophylactic) of nausea and/or emesis following opioid administration. This was a multinational, multicentre, double-blind, parallel group study in which cancer patients who were receiving opioids for cancer pain were randomised to receive oral ondansetron 24 mg once daily, metoclopramide 10 mg three times daily, or placebo. No statistically significant reduction of nausea for any group could be found [27]. Another study in non-cancer patients found an anti-emetic effect of midazolam added to morphine patient-controlled analgesia in women after total abdominal hysterectomy [28]. This study was designed to compare the effect of midazolam to that of ondansetron for prevention of nausea and vomiting during morphine patient-controlled analgesia. Patients were assessed for the incidence of nausea and vomiting, the degree of sedation (awake, mild, moderate, deep) and other side-effects during the first 24 h after the operation. The frequency of nausea and vomiting was significantly lower with midazolam and ondansetron compared with placebo [28]. Two systematic reviews on treatment of OINV could state only weak recommendations for the management of opioid-induced nausea and vomiting. The authors recommended opioid rotation when OINV occurred, but did not recommend the use of antiemetics. The authors proposed a need for high-quality studies before strong recommendations on the management of opioid-induced nausea and vomiting can be made [29, 30].

In contrast to guidelines on treatment of cancer pain, which recommend several different non-opioids, the guidelines for treatment of OINV essentially suggest using antidopaminergic drugs, but are heterogenous about which antidopaminergic drug is mentioned first: metoclopramide or haloperidol (Table 2). This could explain some of the heterogeneity of prescription practice between physicians from specialist and general PC.

### Limitations

There was no structured approach according to Cherrie’s criteria for online surveys [31]. Thus, it is unclear how many colleagues were reached by the inquiry and how many actually responded. A certain bias cannot be excluded, since the survey was initiated via personal contacts and forwarded by this way. Therefore, there is no claim of transferability to the entirety of the medical profession, also because physician’s were probably mostly from Germany and Switzerland. Nevertheless, this is, to the best of our knowledge, one of the first studies to pick up such clear differences in the core questions on the treatment of symptoms. Irrespective of this, further evidence should urgently be obtained against this background.

## Conclusion

Physician’s practice in palliative care for the treatment of cancer pain and OINV differed substantially. Respondents expressed the lack of high-quality evidence-based information from RCTs. Despite all efforts, controlled clinical studies on pharmacological interventions in palliative care are rare, although they are demanded by many important stakeholders and their findings should primarily serve patient safety. We propose that RCTs are needed to inform physicians about benefits and harms of pharmacological treatments of common symptoms in PC. This may help to homogenise guidelines across countries and different institutions. 

### Supplementary Information

Below is the link to the electronic supplementary material.

Supplementary file1 (DOCX 18 KB)

## Data Availability

The data that support the findings of this study are available from the corresponding author [CB] upon reasonable request.
