# Supplementary material for: Pharmacological treatment of cancer pain and opioid induced nausea and vomiting: online survey and comparison with current guidelines
Source: Support Care Cancer. 2024 Jun 15;32(7):436. doi: 10.1007/s00520-024-08628-7 (PMC11180006; doi:10.1007/s00520-024-08628-7)
Supplement: Supplementary file 1 — Supplementary file1 (DOCX 18 KB) [file 520_2024_8628_MOESM1_ESM.docx]

**Supplement “Pharmacological treatment of pain and opioid induced nausea and vomiting: online survey and comparison with current guidelines”**

**Online Questionnaire**

Dear participant. With this survey, we aim to describe the pharmacologic treatment routines for patients with cancer pain, breathlessness and risk of opioid-induced nausea and vomiting.

By continuing, you agree that your anonymous answers are be stored and evaluated by our research group.

We thank you very much for your valuable time (you will need max. 7 minutes to complete the survey). Do not hesitate to contact us in case of questions.

On behalf of the group

Jan Gärtner

University of Basel, Switzerland

[jan.gaertner@pzhi.ch](mailto:jan.gaertner@pzhi.ch)

**CANCER PAIN**

Do you prescribe a non-opioid (i.e. ibuprofen, dipyrone (metamizole), acetylsalicylic acid (ASA/ Aspirin®), paracetamol (acetaminophen), Cox-2 Inhibitors etc) to a patient with moderate to severe cancer pain treated with opioids:

1. Yes, regularly / routinely?
2. Sometimes
3. Rather the exception
4. Never?

If you routinely or sometimes add a non-opioid, what is your first choice:

1. Ibuprofen or other non-steroid-antiphlogistic drugs (NSAID)
2. Paracetamol (Acetaminophen)
3. A Cox-2 Inhibitor
4. Aspirin
5. Dipyrone (Metamizole)

Any comments here: [free text]

My above treatment choices (Cancer Pain), are mainly based on:

1. Intuition and experience
2. Inhouse standards
3. National or international guidelines
4. Randomised controlled trials, meta-analyses
5. Unsure

Any comments here: [free text]

**PREVENTION OF OPIOID INDUCED NAUSEA AND VOMITING**

In patients where you initiate opioid therapy (WHO III). What is your first choice in preventing nausea and vomiting:

1. I prescribe an anti-emetic on demand
2. I prescribe the anti-emetic to be taken regularly (around the clock) for a few days
3. Other

What do you prescribe first choice for the prevention of opioid induced nausea and vomiting:

1. Haloperidol or other neuroleptics
2. Metoclopramide or
3. Antihistamines
4. 5-HT3 Antagonists
5. NK-1 Inhibitors
6. Other

Any comments here: [free text]

My above treatment choices (anti-emetics), are mainly based on:

1. Intuition and experience
2. Inhouse standards
3. National or international guidelines
4. Randomised controlled trials, meta-analyses
5. Unsure

Any comments here: [free text]

**SOCIODEMOGRAPHIC AND OCCUPATIONAL CHARACTERISTICS OF PARTICIPANTS**

My profession:

I am:

1. A physician
2. A nurse
3. Other

My main discipline is best described as

1. Specialist Palliative Care
2. Oncology
3. Geriatric Oncology
4. Cardiology
5. Internal Medicine
6. General practitioner
7. other

I have experience in the above field for

1. less than 5 years
2. 5-10 years
3. More than 10 years

I work in:

1. Europe
2. Latin-America
3. North-America
4. Africa
5. Australia
6. Asia

If you work in Europe, do you work in (please choose according to your personal judgment)

1. Scandinavia
2. UK
3. Central Europe
4. Southern Europe
5. Eastern Europe

Any other comments from your side here: [free text]

We thank you very much for your time and help!

In case you have any questions or want to get in contact, please feel free to contact us:

Jan.gaertner@pzhi.ch
